# Esomeprazole and aspirin in Barrett's oesophagus (AspECT): a randomised factorial trial

**DOI:** 10.1016/S0140-6736(18)31388-6

**Published:** 2018-08-04

**Authors:** Janusz A Z Jankowski, John de Caestecker, Sharon B Love, Gavin Reilly, Peter Watson, Scott Sanders, Yeng Ang, Danielle Morris, Pradeep Bhandari, Stephen Attwood, Krish Ragunath, Bashir Rameh, Grant Fullarton, Art Tucker, Ian Penman, Colin Rodgers, James Neale, Claire Brooks, Adelyn Wise, Stephen Jones, Nicholas Church, Michael Gibbons, David Johnston, Kishor Vaidya, Mark Anderson, Sherzad Balata, Gareth Davies, William Dickey, Andrew Goddard, Cathryn Edwards, Stephen Gore, Chris Haigh, Timothy Harding, Peter Isaacs, Lucina Jackson, Thomas Lee, Peik Loon Lim, Christopher Macdonald, Philip Mairs, James McLoughlin, David Monk, Andrew Murdock, Iain Murray, Sean Preston, Stirling Pugh, Howard Smart, Ashraf Soliman, John Todd, Graham Turner, Joy Worthingon, Rebecca Harrison, Hugh Barr, Paul Moayyedi

**Affiliations:** a Gastroenterology Unit, Morecambe Bay University Hospitals NHS Trust, Lancaster, UK; b National Institute for Health and Care Excellence, London, UK; c Royal College of Surgeons in Ireland, Dublin, Ireland; d Digestive Diseases Centre, University Hospitals of Leicester, Leicester, UK; e College of Medicine, Biological Sciences and Psychology, University of Leicester, Leicester, UK; f Department of Pathology, University Hospitals of Leicester, Leicester, UK; g Centre for Statistics in Medicine, University of Oxford, Oxford, UK; h MRC Clinical Trials Unit at University College London, London, UK; i Queens University, Belfast, UK; j South Warwickshire NHS Foundation Trust, Warwick, UK; k Wrightington, Wigan & Leigh NHS Foundation Trust, Wigan, UK; l GI Science, Salford Royal NHS Foundation Trust and University of Manchester, Manchester, UK; m Queen Elizabeth II Hospital, Welwyn Garden City, UK; n Queen Alexandra Hospital, Portsmouth, UK; o School of Medicine, Pharmacy and Health, Durham University, Durham, UK; p Nottingham Digestive Diseases Centre & BRC, University of Nottingham, UK; q Royal Oldham Hospital, Oldham, UK; r North Manchester General Hospital, Manchester, UK; s Gartnavel General Hospital & Glasgow Royal Infirmary, Glasgow, UK; t Barts Health NHS Trust, London, UK; u Western General Hospital & Royal Infirmary Edinburgh, Edinburgh, UK; v Antrim Area Hospital, Antrim, UK; w Torbay and South Devon NHS Foundation Trust, Torquay, UK; x Oncology Clinical Trials Office, University of Oxford, Oxford, UK; y Queen Margaret Hospital, Dunfermline, UK; z Victoria Hospital, Kirkcaldy, UK; aa Craigavon Area Hospital, Portadown, UK; ab Ninewells Hospital, Dundee, UK; ac Birmingham City Hospital, Birmingham, UK; ad Harrogate District Hospital, Harrogate, UK; ae Altnagelvin Area Hospital, Londonderry, UK; af Royal Derby Hospital, Derby, UK; ag Yeovil District Hospital, Yeovil, UK; ah Wansbeck General Hospital, Ashington, UK; ai Lagan Valley Hospital, Lisburn, UK; aj Royal Cornwall Hospital, Truro, UK; ak North Tyneside General Hospital, North Shields, UK; al Mater Infirmorum Hospital, Belfast, UK; am Cumberland Infirmary, Carlisle, UK; an Darent Valley Hospital, Dartford, UK; ao Countess of Chester Hospital, Chester, UK; ap St Bartholomew's Hospital, London, UK; aq Musgrove Park Hospital, Somerset, UK; ar Royal Liverpool University Hospital, Liverpool, UK; as Barnsley Hospital NHS Foundation Trust, Barnsley, UK; at Gloucester Royal Hospital, Gloucester, UK; au Department of Medicine, McMaster University Ontario, Hamilton, ON, Canada; av Blackpool Victoria Hospital, Blackpool, UK

## Abstract

**Background:**

Oesophageal adenocarcinoma is the sixth most common cause of cancer death worldwide and Barrett's oesophagus is the biggest risk factor. We aimed to evaluate the efficacy of high-dose esomeprazole proton-pump inhibitor (PPI) and aspirin for improving outcomes in patients with Barrett's oesophagus.

**Methods:**

The Aspirin and Esomeprazole Chemoprevention in Barrett's metaplasia Trial had a 2 × 2 factorial design and was done at 84 centres in the UK and one in Canada. Patients with Barrett's oesophagus of 1 cm or more were randomised 1:1:1:1 using a computer-generated schedule held in a central trials unit to receive high-dose (40 mg twice-daily) or low-dose (20 mg once-daily) PPI, with or without aspirin (300 mg per day in the UK, 325 mg per day in Canada) for at least 8 years, in an unblinded manner. Reporting pathologists were masked to treatment allocation. The primary composite endpoint was time to all-cause mortality, oesophageal adenocarcinoma, or high-grade dysplasia, which was analysed with accelerated failure time modelling adjusted for minimisation factors (age, Barrett's oesophagus length, intestinal metaplasia) in all patients in the intention-to-treat population. This trial is registered with EudraCT, number 2004-003836-77.

**Findings:**

Between March 10, 2005, and March 1, 2009, 2557 patients were recruited. 705 patients were assigned to low-dose PPI and no aspirin, 704 to high-dose PPI and no aspirin, 571 to low-dose PPI and aspirin, and 577 to high-dose PPI and aspirin. Median follow-up and treatment duration was 8·9 years (IQR 8·2–9·8), and we collected 20 095 follow-up years and 99·9% of planned data. 313 primary events occurred. High-dose PPI (139 events in 1270 patients) was superior to low-dose PPI (174 events in 1265 patients; time ratio [TR] 1·27, 95% CI 1·01–1·58, p=0·038). Aspirin (127 events in 1138 patients) was not significantly better than no aspirin (154 events in 1142 patients; TR 1·24, 0·98–1·57, p=0·068). If patients using non-steroidal anti-inflammatory drugs were censored at the time of first use, aspirin was significantly better than no aspirin (TR 1·29, 1·01–1·66, p=0·043; n=2236). Combining high-dose PPI with aspirin had the strongest effect compared with low-dose PPI without aspirin (TR 1·59, 1·14–2·23, p=0·0068). The numbers needed to treat were 34 for PPI and 43 for aspirin. Only 28 (1%) participants reported study-treatment-related serious adverse events.

**Interpretation:**

High-dose PPI and aspirin chemoprevention therapy, especially in combination, significantly and safely improved outcomes in patients with Barrett's oesophagus.

**Funding:**

Cancer Research UK, AstraZeneca, Wellcome Trust, and Health Technology Assessment.

## Introduction

The incidence of oesophageal adenocarcinoma has increased substantially in North America and Europe over the past 40 years.^1^ Although incidence might be plateauing, areas such as Hawaii are still seeing annual increases of 8%.^1^ There are over 52 000 cases of oesophageal adenocarcinoma worldwide each year and 5-year survival is less than 10% when detected through symptoms. Increasing incidence of oesophageal adenocarcinoma is probably related to the rise in gastro-oesophageal reflux disease in high-income countries, especially in populations of European descent.^2^, ^3^, ^4^, ^5^

Gastro-oesophageal reflux is one of the main risk factors for Barrett's oesophagus, in which a portion of the oesophagus that is usually lined with squamous epithelium undergoes metaplastic change to become columnar mucosa. Barrett's oesophagus is a complex, genetically predisposed, premalignant condition^6^ that affects 2% of the adult population in western countries and can progress to adenocarcinoma, following the sequence oesophagitis-metaplasia-dysplasia-adenocarcinoma.^7^, ^8^ Surveillance of Barrett's oesophagus to detect early stage cancer has been associated with only a modest improvement in the outlook of oesophageal adenocarcinoma.^9^ Strategies to prevent progression to oesophageal adenocarcinoma could have a substantial impact in the same way that screening for colorectal cancer has proved successful in reducing colorectal cancer deaths.^10^

**Research in context**

**Evidence before this study**

We did a very large systematic review of the world literature using a Delphi process, which has been published previously (Bennett C, et al. *Am J Gastroenterol* 2015; **110:** 662–82). This search covered more than 20 000 papers in English, with hand searching of over 500 other non-English papers. We designed the scope, proposed statements, and searched electronic databases resulting in 20 558 papers that were screened, selected online, and formed our evidence base. We used a Delphi consensus with an 80% agreement threshold using Grading of Recommendations Assessment, Development and Evaluation assessment to determine the quality of the evidence. We found no significant evidence to recommend the use of proton-pump inhibitors (PPIs) and low-dose aspirin for prophylaxis in Barrett's oesophagus. The review has been badged as formal NICE and NHS Evidence because of its rigour.

**Added value of this study**

To our knowledge, AspECT is the largest randomised controlled trial of PPIs and aspirin in Barrett's oesophagus of any kind and the largest chemoprevention trial of PPIs. It is one of the longest aspirin chemoprevention trials with more than 20 000 patient-years of follow up. It is also unique in that it has assessed the combination of two successful chemoprevention agents.

**Implications of all the available evidence**

High-dose PPI therapy (80 mg esomeprazole per day) prolonged time to the composite endpoint of all-cause mortality, oesophageal adenocarcinoma, and high-grade dysplasia in patients with Barrett's oesophagus compared with low-dose PPI (20 mg per day). Aspirin also had an effect on these endpoints, but this was only significant when patients who received non-steroidal anti-inflammatory drugs were censored from the analysis. Both treatments seemed to have an additive effect. Clinically significant side-effects were rare.

Early detection of Barrett's oesophagus is confined to research settings; however, there are promising chemoprevention strategies. Proton pump inhibitors (PPIs) effectively reduce acid reflux, which is thought to be one of the main drivers of Barrett's oesophagus. After the development of Barrett's oesophagus, PPIs downregulate cylcogoxygenase-2 expression, which might protect against neoplastic progression.^11^ Observational data have suggested that patients with Barrett's oesophagus who are taking PPIs have reduced neoplastic progression,^12^ but this is low-quality, controversial evidence.^13^ A systematic review from 2014 supports the view that powerful acid suppression could reduce risk of neoplasia.^14^ Esomeprazole is the most commonly used PPI in the USA, and allows the healing of oesophagitis without promoting clonal expansion of Barrett's oesophagus.^15^ Observational data suggest that aspirin use is associated with reduced risk of oesophageal adenocarcinoma,^16^, ^17^, ^18^, ^19^ but this is not a universal finding.^20^ Finally, although Barrett's oesophagus is a major risk factor for oesophageal adenocarcinoma, only a minority of patients with Barrett's oesophagus die from oesophageal adenocarcinoma; most die from cardiovascular disease or chest infections.^21^ Preventive strategies should ideally affect overall mortality.

No randomised trial has so far evaluated PPIs or aspirin for improving outcomes, including preventing neoplastic progression, in patients with Barrett's oesophagus. We aimed to evaluate the efficacy and safety of these agents in the Aspirin and Esomeprazole Chemoprevention in Barrett's metaplasia Trial (AspECT), especially their ability to reduce all-cause mortality, oesophageal adenocarcinoma, and high-grade dysplasia.

## Methods

### Study design and participants

AspECT is a phase 3, randomised prospective factorial study of chemoprevention by aspirin and esomeprazole in patients with Barrett's oesophagus. There were 84 centres across England, Scotland, Wales, and Northern Ireland, and one in McMaster Health Sciences Centre, Hamilton, ON, Canada. AspECT was approved by the Main Research Ethics Committee in the UK (REC reference P1/04/Q0603/1) and by the Hamilton Integrated Research Ethics Board in Canada (reference 06-2731). All participants provided fully informed written consent.

Participants were recruited by gastroenterologists and upper gastrointestinal surgeons through hospital clinics and endoscopy lists, including new and existing Barrett's oesophagus diagnoses. Individuals aged 18 years or older meeting the globally accepted criteria for Barrett's oesophagus, at least 1 cm of histologically proven columnar-lined oesophagus,^22^ were eligible to participate. Exclusion criteria included pre-existing oesophageal adenocarcinoma, high-grade dysplasia, or taking non-steroidal anti-inflammatory drugs (NSAIDs) at baseline. Detailed inclusion and exclusion criteria are provided in the appendix. Because women with Barrett's oesophagus have a lower risk of oesophageal adenocarcinoma than do men,^22^ we limited recruitment of women to approximately 500.

### Randomisation and masking

Participants were randomised 1:1:1:1 in a 2 × 2 factorial design to receive esomeprazole at either high or low dose, with or without aspirin. Randomisation was done using a computer-generated schedule administered by a central trials unit to maintain allocation concealment. Some patients had contraindications to or were already taking aspirin for cardiovascular secondary prevention. We allowed these participants to enter PPI randomisation only. We therefore expected more participants in the PPI than the aspirin randomisation. Randomisation was by minimisation with a random element of 0·8. The minimisation factors chosen were possible risk factors for the development of high-grade dysplasia, adenocarcinoma, and death: length of Barrett's oesophagus (tongue or <2 cm or ≥2 cm, ≤3 cm or >3 cm, and ≤8 cm or >8 cm), age (18–49, 50–59, 60–69, ≥70 years), and intestinal metaplasia (yes or no). Using minimisation with the same variables, women and men were randomly assigned to treatment separately, as were those participants who only took part in the PPI randomisation. Treatment was not blinded.

### Procedures

Patients received esomeprazole at either a high dose (40 mg capsules twice-daily) or a low dose (20 mg capsules once-daily). Patients who were assigned aspirin received one standard-dose tablet per day (300 mg in the UK, 325 mg in Canada). At follow-up visits, which occurred once per year starting from enrolment (±3 months), all patients were asked about hospital admissions and medical records were checked for serious adverse events. Follow-up in years 1, 3, 5, 7, and 9 was by face-to-face or telephone interview, and in years 2, 4, 6, 8, and 10, patients underwent endoscopy. All centres were trained and centrally monitored for endoscopy and pathology quality: strict adherence was essential for both site set up and for individual participant recruitment, with inclusion criteria being validated by a trial office using faxed or scanned endoscopy and pathology forms before enrolment.

Trial endoscopists received training in the use of the Prague C and M criteria for diagnosis and grading of Barrett's oesophagus with central monitoring of images and videos.^23^ Standardised pathology criteria for reporting Barrett's oesophagus biopsies were developed, with training overseen by a central pathology panel as reported previously.^24^ At each endoscopy, four-quadrant biopsies were taken every 2 cm along the areas of Barrett's oesophagus, with separate targeted biopsy of any macroscopic abnormalities. Biopsies were fixed in buffered formalin, transported to the pathology lab, processed within 24 h, embedded in wax, cut, stained with haematoxylin and eosin and assessed by local gastrointestinal pathologists. All pathology diagnoses of high grade dysplasia and adenocarcinoma were double reported by a second pathologist at trial centres (RCPath best practice). A 10% audit of all trial centre pathology diagnoses, including high proportions of high grade dysplasia and adenocarcinoma cases, was undertaken by a central trial pathology review panel^24^ with complete agreement for cases of high grade dysplasia and adenocarcinoma. Reports were seen by the local clinical team and decisions were implemented, then faxed to the central trial office for validation and checking. All centres in all countries adhered to the same protocol except for the differing doses of aspirin between the UK and Canada.

### Outcomes

The co-primary aims were the efficacy of high-dose PPI versus low-dose PPI, and the efficacy of aspirin versus no aspirin. The primary composite endpoint was time to the all-cause mortality, oesophageal adenocarcinoma, or high-grade dysplasia, whichever occurred first. Secondary aims (which were not fully powered) included each treatment's effect on time to the individual components of the composite endpoint, cause-specific mortality, and the composite endpoint analysed by sex. Secondary outcomes that are not reported in this Article were molecular risk factors in Barrett's oesophagus for the development of oesophageal adenocarcinoma, the cost effectiveness of aspirin and PPI treatment, whether intervention with PPI or aspirin induces changes in the expression of molecular markers for oesophageal adenocarcinoma, the genomics of aspirin sensitivity, how quality of life is affected by the different treatments, and the biological risk factors for cardiac disease and aspirin resistance.

### Statistical analysis

We used intention-to-treat analysis for all efficacy analyses, analysing all participants who underwent randomisation and did not rescind consent in the treatment groups they were assigned to. We checked the significance of the treatment interaction term by first adding an interaction term to a primary model before using at-the-margins and within-table results to produce an interaction ratio. While we recognise that the power was low for this interaction comparison, we concluded that an analysis using the factorial design was appropriate.

All analyses used accelerated failure time (AFT) modelling, with adjustment for minimisation factors. The accelerated failure time models were interpreted in terms of the time to an event using the time ratio (TR). A TR greater than 1 for the composite endpoint implied that the treatment prolonged the time to an event. We used AFT because of the intuitive nature of the TR, which models survival time, its benefit being that results are reported as a delay to an event over the entire trial period compared with the hazard ratio result, which is interpreted as risk of an event at any one given time. Cox proportional hazards survival analyses, and where appropriate, Cox competing risks survival analyses were also performed on all comparisons to allow for comparison with other research. Before the use of both AFT and Cox survival models, the assumption of proportional hazards was tested with Schoenfeld tests and plots of residuals.

Median follow-up was calculated using a reverse Kaplan-Meier method.^25^

The trial aimed to recruit 5000 participants (1250 in each intervention group), assuming there was no interaction between the effects of aspirin and PPI interventions, an exponential time-to-composite-event with a constant event rate of 0·76% per year, a composite event hazard ratio of 1·4, recruitment over 2 years, follow-up for 8 years, 10% loss to follow-up, 20% non-compliance with medication, 80% power, and a 2-sided test at 5% significance. In October, 2008, at the trial steering committee, data safety and monitoring committee, and funder's request, sample size was amended to allow emerging external data to be incorporated into the statistical calculations, namely published evidence showing a larger effect of aspirin (the raw data was available pre-publication with permission because JJ was a co-author)^27^ and a higher conversion rate to cancer than previously expected, and the realisation that the initial composite event rate was too cautious.^21^ It was agreed that it would be more efficient and cost-effective to decrease the recruitment target but extend follow-up to 10 years to allow more events to accrue in the ageing trial population. The new sample size of 2224 participants (196 events) was based on the original calculations, but with amendments to the constant event rate for the composite event (death, cancer or high-grade dysplasia) to a conservative 1% per year, the composite event hazard ratio to 1·5, recruitment to 3 years, follow-up for a maximum of 10 years, and the adjustment for medication compliance removed. With the agreement of the trial steering committee and data safety and monitoring committee, the funder permitted the trial to recruit until the end of February, 2009, or until 2224 participants, had been accrued, whichever was later.

The primary outcome was analysed and presented confidentially to the trial's data safety monitoring committee as specified in the protocol after 2 and 4 years of follow-up as interim analyses, with a p value less than or equal to 0·001 regarded as significant. The committee recommended trial continuation and neither interim analysis was disseminated further.

UK sites also collected information on NSAID use. As specified in the statistical analysis plan, we did an analysis including only UK participants and censored follow-up when a participant began taking NSAIDs. We could then compare aspirin use with no aspirin, in the absence of NSAIDs.

The 2 × 2 factorial design provides two co-primary comparisons, high-dose PPI compared with low-dose PPI, and aspirin compared with no aspirin. Secondary analyses of each element of the composite endpoint (high-grade dysplasia, oesophageal adenocarcinoma, all-cause mortality) were evaluated in the same way as the primary comparisons using both AFT and Cox survival analyses. A per-protocol population was defined based on treatment and trial compliance, as detailed in the appendix, with all analyses repeated as per primary methods. There were no missing data present in variables used in the primary and secondary analyses. No adjustments were made to any analyses for multiple testing. Number needed to treat (NNT) and number needed to harm were calculated as one divided by the absolute risk difference of the primary event or adverse event, respectively. Safety data are presented in descriptive form without further statistical analysis. All analyses were done with Stata version 15.0.

### Role of the funding source

The funder of the study had no role in study design, data collection, data analysis, data interpretation, or writing of the report. JdC, JAZJ, YA, SA, SBL, RH, DMor, HB, SS, PW, AW, CB, GR, PB, and PMo had full access to all the data in the study and JAZJ, PMo, SL, GR, JdC, HB, SS, RH, and CB had final responsibility for the decision to submit for publication.

## Results

**Figure 1:**
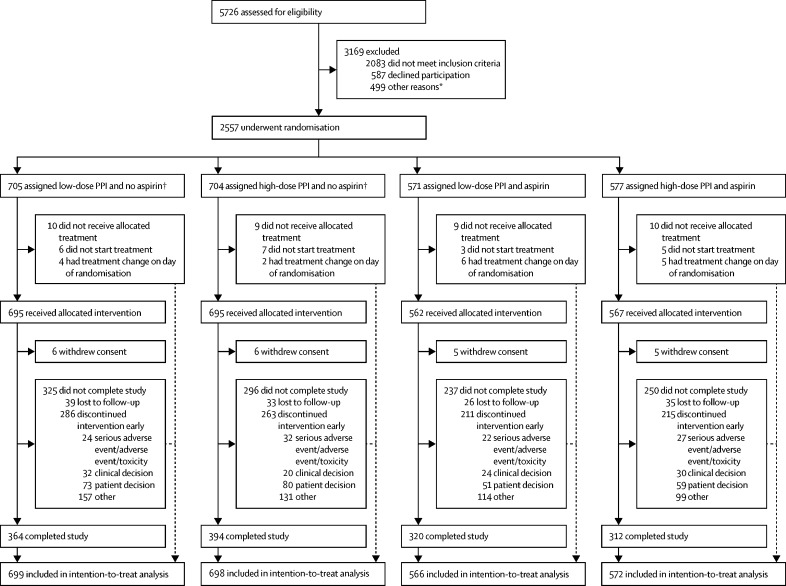
Trial profile The intention to treat analysis included all patients who underwent randomisation, with the eception of those who withdrew consent to the use of their data. PPI=proton-pump inhibitor. *Details of exclusions for other reasons are available in the appendix. †255 patients were randomised to the PPI groups only.

We recruited 2557 patients with Barrett's oesophagus from March 10, 2005, to March 1, 2009, and followed them up for a median of 8·9 years (IQR 8·2–9·8), collecting 20 095 patient-years of data. The number of patients recruited, 2557, was 15% over the minimum power needed. 705 patients were assigned to low-PPI and no aspirin, 704 to high-PPI and no aspirin, 571 to low-PPI and aspirin, and 577 to high-PPI and aspirin (figure 1). 313 primary endpoint events occurred. Follow-up was completed by March 1, 2017 (figure 1; appendix). Baseline characteristics are shown in table 1 and the appendix and compliance with medication is shown in the appendix. The trial achieved a data return rate of 99·9%, with only one case report form outstanding out of 66 200. Intestinal metaplasia was found in 2266 (89%) patients at initial endoscopy, with the remainder having a mosaic of gastric metaplasia, increasing to 100% with intestinal metaplasia on subsequent endoscopies.^24^, ^26^

**Table 1:** Baseline characteristics by treatment group

		**Low-dose PPI (n=1265)**	**High-dose PPI (n=1270)**	**No aspirin (n=1142)**	**Aspirin (n=1138)**
Length of Barrett's metaplasia at randomisation (strata for minimisation, cm)*		4 (3–6)	4 (2–6)	4 (2–6)	4 (3–6)
Length of Barrett's oesophagus (stratification group, cm)					
	<2	123 (10%)	124 (10%)	108 (9%)	109 (10%)
	2–3	434 (34%)	435 (34%)	398 (35%)	395 (35%)
	3–8	538 (43%)	539 (42%)	491 (43%)	493 (43%)
	>8	130 (10%)	129 (10%)	117 (10%)	118 (10%)
	Tongues	40 (3%)	43 (3%)	28 (2%)	23 (2%)
Age (strata for minimisation, years)		59 (51–65)	59 (51–65)	58 (50–64)	58 (50–65)
Age (stratification grouping, years)					
	<50	283 (22%)	280 (22%)	269 (24%)	272 (24%)
	50–60	388 (31%)	390 (31%)	365 (32%)	358 (31%)
	60–70	447 (35%)	445 (35%)	386 (34%)	388 (34%)
	>70	147 (12%)	155 (12%)	122 (11%)	122 (11%)
Intestinal metaplasia					
	Yes	1130 (89%)	1136 (89%)	1042 (91%)	1035 (91%)
	No	134 (11%)	134 (11%)	100 (9%)	103 (9%)
Sex					
	Male	1012 (80%)	1010 (80%)	900 (79%)	896 (79%)
	Female	253 (20%)	260 (20%)	242 (21%)	242 (21%)

The length of Barrett's oesphagus stratification group was required for randomisation. The actual length of Barrett's oesphagus was collected on the baseline data form. PPI=proton-pump inhibitor (esomeprazole).

* Data missing from 122 patients.

The PPI-aspirin interaction term was not significant (p=0·2807, n=2280, TR 1·30, 95% CI 0·81–2·09), so we analysed the PPI and aspirin comparisons separately. Event rates in each group are shown in the appendix.

**Figure 2:**
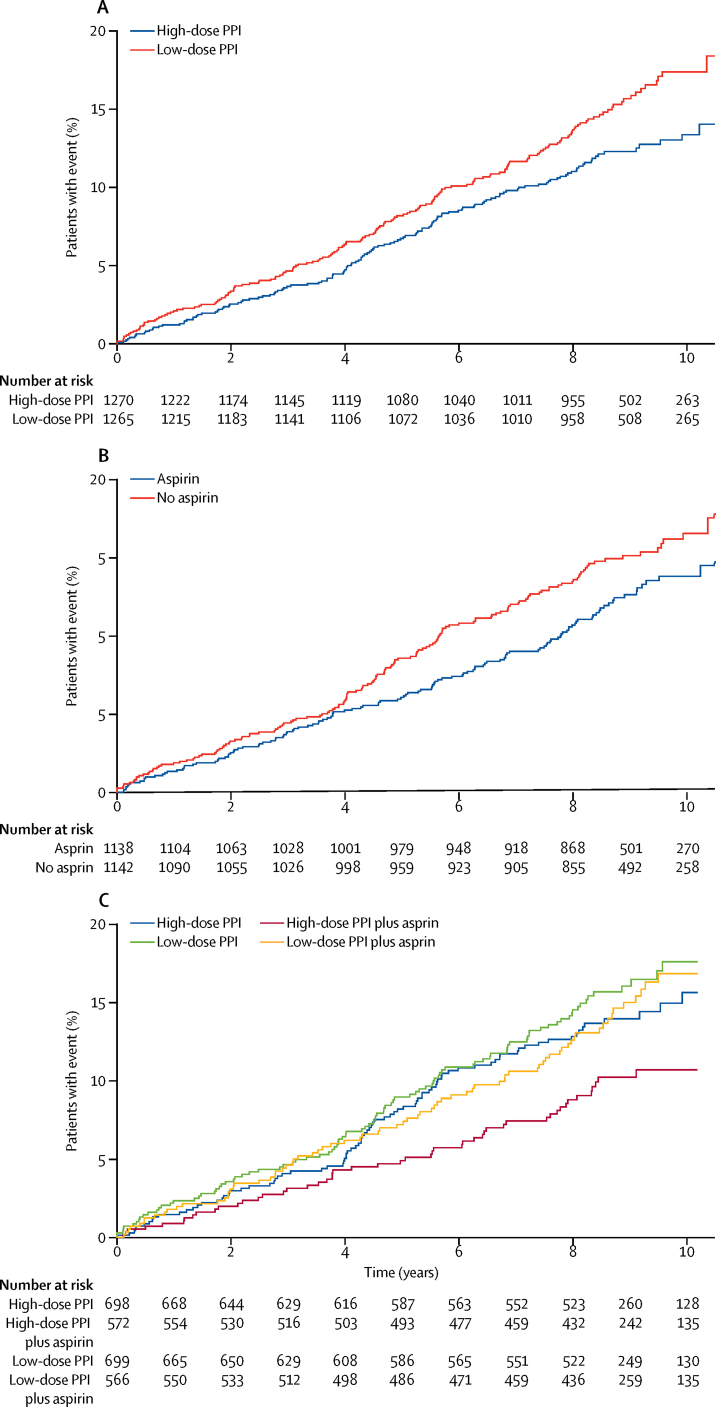
Event-free survival Curves show survival until the composite endpoint events (high-grade dysplasia, oesophageal adenocarcinoma, all-cause mortality) in the (A) high-dose PPI and low-dose PPI groups, (B) the aspirin and no aspirin groups, and (C) all four treatment groups. PPI=proton-pump inhibitor.

In the primary analysis for PPI (figure 2A), the high dose (139 events in 1270 patients) was significantly more effective than the low dose (174 events in 1265 patients; TR 1·27, 95% CI 1·01–1·58, p=0·038). High-dose PPI significantly lengthened the time to reach outcome events, suggesting that high-dose PPI delays death, cancer, and high-grade dysplasia. If the expected time to the composite event while taking low-dose PPI was 8 years (the original expected duration of treatment), taking high-dose PPI would increase this to 10·2 years (95% CI 8·1–12·6).

In the primary analysis for aspirin (figure 2B), the effect of aspirin (127 events in 1138 patients) was not significantly different from that with no aspirin (154 events in 1142 patients; TR 1·24, 95% CI 0·98–1·57, p=0·068). In this subgroup, aspirin (125 events in 1116 patients) was significantly better than no aspirin (150 events in 1120 patients) for the composite endpoint when not combined with NSAIDs (TR=1·29, 95% CI 1·01–1·66, p=0·043).

The effects of PPI and aspirin seemed to be additive when taken in combination (figure 2C). The largest difference was in the comparison of combined aspirin and high-dose PPI (52 events in 572 participants) with low-dose PPI and no aspirin (99 events in 699 participants; TR 1·59, 95% CI 1·14–2·23, p=0·0068). We also compared the effect of aspirin combined with high-dose PPI (52 events in 572 patients) with that of high-dose PPI alone (87 events in 698 patients), which gave a TR to endpoint of 1·38 (0·98–1·94, p=0·0680). The confidence interval suggests support for this effect, although it was not statistically significant as the trial was not powered for this analysis.

Table 2 shows the results of the secondary analyses. High-dose PPI decreased all-cause mortality compared with low-dose PPI. For high-grade dysplasia (the precursor lesion to oesophageal adenocarcinoma), the comparison of aspirin versus no aspirin gave a TR of 1·51 (95% CI 1·00–2·29, p=0·053).

**Table 2:** Accelerated failure time modelling for secondary endpoints

	**High-dose PPI *vs* low-dose PPI**					**Aspirin *vs* no aspirin**				
	Total number of patients in analysis	Events/patients on high-dose PPI	Events/patients on low-dose PPI	Time ratio (95% CI)	p value	Total number of patients in analysis	Events/patients on aspirin	Events/patients not on aspirin	Time ratio (95% CI)	p value
All-cause mortality	2535	79/1270	105/1265	1·36 (1·01–1·82)	0·039	2280	73/1138	90/1142	1·25 (0·92–1·70)	0·16
Oesophageal adenocarcinoma	2535	40/1270	41/1265	1·04 (0·67–1·61)	0·86	2280	35/1138	35/1142	1·02 (0·64–1·64)	0·92
High-grade dysplasia	2535	44/1270	59/1265	1·36 (0·92–2·02)	0·12	2280	37/1138	55/1142	1·51 (1·00–2·29)	0·053
Cause-specific mortality	2535	8/1270	12/1265	1·55 (0·63–3·80)	0·34	2280	8/1138	8/1142	1·01 (0·38–2·69)	0·98
Composite endpoint, men only	2022	118/1010	148/1012	1·26 (0·99–1·61)	0·06	1796	105/896	130/900	1·26 (0·98–1·64)	0·07
Composite endpoint, women only	513	21/260	26/253	1·27 (0·72–2·27)	0·41	484	22/242	24/242	1·13 (0·63–2·02)	0·69

PPI=proton pump inhibitor (esomeprazole).

We designed the trial to use accelerated failure time modelling and give TRs, because these are easier to interpret than other estimates. The appendix includes the results from a Cox model with hazard ratios to allow for comparison with the results of other studies. The appendix also includes Kaplan Meier plots for effects on all-cause mortality and high-grade dysplasia or oesophageal adenocarcinoma separately, respectively, for aspirin versus no aspirin and high-dose PPI versus low-dose PPI. In the secondary analysis, PPIs had a significant effect on all-cause mortality.

We calculated the NNT to prevent high-grade dysplasia, adenocarcinoma, or death with both primary therapies (aspirin *vs* no aspirin, low-dose PPI *vs* high-dose PPI). In the aspirin comparison, we estimated that, on average, 43 patients would need to be treated with aspirin to prevent one event (95% CI 20–250). In the PPI comparison, we calculated an NNT of 34 (18–333) for high-dose PPI—ie, 34 patients would need to be treated with high-dose PPI instead of low-dose PPI to prevent one event.

1132 serious adverse events occurred in 718 participants, of which 65 serious adverse events in 61 participants were considered to be related to one or both treatments. Those with Common Terminology Criteria for Adverse Events grade 3–5 events are shown in table 3. Only 28 (1%) participants had a serious adverse event of grade 3–5 that was related to a study treatment (table 3; appendix). 64 episodes of haemorrhage were recorded in 59 patients, with more events in the aspirin groups (38 patients receiving aspirin and 21 patients not receiving aspirin), but less than 1% (20 patients) of all patients experienced a grade 3–5 bleed. Seven grade 3–5 gastrointestinal bleeds were reported across seven patients (appendix). In total, there were 303 serious adverse events among the 704 patients receiving high-dose PPI versus 274 events among the 571 patients receiving high-dose PPI and aspirin combination, with little difference in the proportion of patients with severe adverse events between the groups. Of the most severe grade 3–5 adverse events, there were 15 related to aspirin and 13 related to PPI.

**Table 3:** Serious adverse events and serious adverse reactions

	**PPI**		**Aspirin**	
	Low-dose PPI (n=1265)	High-dose PPI (n=1270)	No aspirin (n=1142)	Aspirin (n=1138)
**Serious adverse events**				
Blood and lymphatic system disorders	4	3	1	4
Cardiac disorders	57	56	42	53
Ear and labyrinth disorders	1	2	1	2
Endocrine disorders	1	1	1	1
Eye disorders	1	3	1	3
Gastrointestinal disorders	30	28	22	32
General disorders and administration site conditions	7	11	9	8
Hepatobiliary disorders	16	10	12	12
Immune system disorders	1	2	3	0
Infections and infestations	57	66	48	64
Injury, poisoning, and procedural complications	28	23	22	24
Investigations	2	1	1	2
Metabolism and nutrition disorders	2	7	5	2
Musculoskeletal and connective tissue disorders	7	4	4	7
Neoplasms benign, malignant, and unspecified (including cysts and polyps)	56	52	52	41
Nervous system disorders	31	26	25	28
Psychiatric disorders	4	8	4	5
Renal and urinary disorders	7	10	3	8
Respiratory, thoracic, and mediastinal disorders	8	7	4	8
Skin and subcutaneous tissue disorders	0	1	0	0
Vascular disorders	15	14	12	14
Total	335	335	272	318
**Serious adverse reactions**				
Related to aspirin	9	6	0	15
Related to esomeprazole	4	9	8	4
Related to both aspirin and esomeprazole	0	0	0	0
Total	13	15	8	19

Adverse events and reactions shown are those of CTCAE grades 3–5, by treatment group. 19 serious adverse events were missing a CTCAE grade. PPI=proton-pump inhibitor. CTCAE=Common Terminology Criteria for Adverse Events.

## Discussion

To our knowledge, AspECT is the first randomised trial to evaluate PPI and aspirin chemoprevention in Barrett's oesophagus and the largest randomised trial of Barrett's oesophagus ever done, with 20 095 participant-years of follow-up in 2557 patients. We have shown that high-dose PPI use protects against a composite endpoint of all-cause mortality, oesophageal adenocarcinoma, and high-grade dysplasia. Aspirin use also protected against the composite endpoint when patient follow-up was censored at start of concomitant NSAID use. The data suggest the effects of the two therapies are additive, as the group who took both high-dose PPI and aspirin had the strongest benefit. High-dose PPI seemed to confer the single biggest effect, and combination with aspirin added another 38% to the time to an event. Both agents were well-tolerated with few serious adverse events. It seems likely that the use of aspirin and PPI would improve survival in Barrett's oesophagus if given for at least 9 years.

This study has several limitations. Because we assessed only a small fraction of patients with Barrett's oesophagus in predominantly white populations in five countries, our results might not be fully generalisable to all ethnic populations. However, Barrett's oesophagus is currently most common worldwide in white populations. We also limited the study to only about 500 women. Although our drug treatment was not blinded, the outcomes of oesophageal adenocarcinoma and all-cause mortality are objective and unlikely to be biased by lack of blinding, even taking potential placebo or nocebo effects into account. A masked pathology panel with double reporting was used to minimise bias in evaluating high-grade dysplasia and oesophageal adenocarcinoma. 255 patients took part only in the PPI randomisation because they were aspirin intolerant or not able to stop taking aspirin, so, given that this was a non-selected patient group, this is a more generalisable group reflecting the situation in the population at large; since these patients were randomly assigned to low-dose PPI and high-dose PPI, we would not expect an effect on the PPI comparison. The 95% CIs are wide and the lower limit was close to 1 when each drug was evaluated individually, suggesting that the results are not robust. Aspirin and NSAIDs are available over the counter, so participants could have taken these drugs without reporting this to the investigator. However, this would have biased the results towards the null hypothesis and therefore would only underestimate aspirin's efficacy.

Our data are supported by a meta-analysis of selected randomised controlled cardiovascular prevention trials evaluating aspirin versus placebo, which found that oesophageal adenocarcinoma was reduced in participants taking aspirin.^17^ There are, however, concerns about these data,^18^ and the studies included in the meta-analysis did not investigate patients with Barrett's oesophagus. Nevertheless, our data add support to the possibility that aspirin prevents oesophageal adenocarcinoma. Although a systematic review of observational studies suggested that PPI therapy reduces the risk of oesophageal adenocarcinoma and high-grade dysplasia,^14^ these results are liable to the bias or confounding inherent to observational studies.

Our results with PPI are supported at the physiological level by studies showing that twice-daily PPI produces more effective suppression of acid reflux than once-daily dosing and, more provocatively, that high-dose PPI also allows preferential healing of Barrett's oesophagus segments into squamous epithelium.^15^, ^28^ There is little data in the literature on combining PPI and aspirin to prevent neoplastic progression of Barrett's oesophagus, and these are the first randomised trial data to suggest the two drugs might have additive effects.

Our results have implications for clinical practice. Current guidelines in the UK and North America for Barrett's and reflux oesophagitis propose that the “lowest effective dose to minimise reflux symptoms should be used”.^22^, ^29^ Our data indicate that high-dose PPI (40 mg twice-daily) is better than low-dose (20 mg once-daily) for patients with Barrett's oesophagus in terms of delaying death, cancer, and dysplasia. Our data also suggest that 300 mg or 325 mg daily aspirin is effective for the composite endpoint, although we do not know if this is the optimal dose. The NNTs for high-dose PPI and aspirin are 34 and 43, respectively, to prevent one event. Combining high-dose PPI and aspirin seems to be more effective in reducing the composite endpoint than either treatment alone. The combination appears safe, with only 1% of participants reporting a serious adverse event of grade 3–5, with little increase in adverse events when adding aspirin to high-dose PPI. Current guidelines do not address the possibility of giving aspirin to reduce neoplastic progression in patients with Barrett's oesophagus; our results suggest that a review of the existing guidelines is warranted.

Several questions remain unanswered. How long must patients take the combination of PPI and aspirin to benefit from any chemopreventative effects on oesophageal stem cells? Before 5 years, neither therapy had a significant benefit,^19^ but that after 8·9 years of follow-up, the effect was significant. We also do not yet know the pharmacogenomics underlying who responds best to either or both of these therapies,^8^ although investigations on this are underway.^30^ These data also raise the possibility that all patients needing long-term PPI to control reflux symptoms might benefit from the co-prescription of aspirin with acid suppression. PPI could reduce the upper gastrointestinal bleeding associated with aspirin whilst the benefits of aspirin remain. This hypothesis should be investigated in large population-based trials.
