# Department of Error

**DOI:** 10.1016/S0140-6736(18)32970-2

**Published:** 2018-12-15

*Jankowski JAZ, de Caestecker J, Love SB, et al. Esomeprazole and aspirin in Barrett's oesophagus (AspECT): a randomised factorial trial.* Lancet *2018; **392:** 400–08—*The address and email of the corresponding author and the affiliation details for Prof JAZ Jankowski have been updated. Additionally, the AspECT Trial Team has been added to the end of the Article. These corrections have been made to the online version as of December 13, 2018.

