# Supplementary material for: Esomeprazole and aspirin in Barrett's oesophagus (AspECT): a randomised factorial trial
Source: Lancet. 2018 Aug 4;392(10145):400–8. doi: 10.1016/S0140-6736(18)31388-6 (PMC6083438; doi:10.1016/S0140-6736(18)31388-6)

THE LANCET

Supplementary appendix

This appendix formed part of the original submission and has been peer reviewed. We post it as supplied by the authors.

Supplement to: Jankowski JAZ, Joh de Caestecker J, Love SB, et al. Esomeprazole and aspirin in Barrett's oesophagus (AspECT): a randomised factorial trial. *Lancet* 2018; published online July 26. http://dx.doi.org/10.1016/S0140-6736(18)31388-6.

Supplementary Data

Supplementary Table 1: AspECT inclusion and exclusion criteria

**Original eligibility criteria**	**Change in criteria, date of change, and further information**	**Final eligibility criteria**
**Inclusion criteria**		
Male aged 40–75 years	Age limit modified in Protocol V6·0 dated 20 Jan 2006, now 18 years and over<br>Up to 500 women entered for generalizability	Aged ≥18 years
Circumferential Barrett's metaplasia at least 2 cm from the gastro-esophageal junction (histologically proven by intestinal metaplasia in at least one sample)	Changed to circumferential Barrett's metaplasia at least 1 cm long in Protocol_V6·0_dated 20Jan2006<br>Added a past history of, but not current, intestinal metaplasia in Protocol_V6·0_dated 20Jan2006<br>Changed in Protocol V9·0 dated 25 Sep 2007 to no need for intestinal metaplasia and allowing non-circumferential tongues of Barrett's esophagus >2 cm as enough for inclusion	Circumferential Barrett's esophagus of at least 1 cm in length (≥C1M1) or a tongue of Barrett's esophagus of at least 2 cm in length (≥C0M2), irrespective of the presence now or historically of histologically proven intestinal metaplasia
Able to give written informed consent	Unchanged	Able to give written informed consent
WHO activity profile of 0 or 1, i.e., fully active and self-caring	Unchanged	WHO activity profile of 0 or 1, i.e., fully active and self-caring
**Exclusion criteria**		
High-grade dysplasia or carcinoma at enrolment	Unchanged	High-grade dysplasia or carcinoma at enrolment

Medical conditions that would make endoscopy or completing the trial difficult, including:<br><br>• Frequent transient ischemic attacks (3 or more) or severe cerebral vascular accident in the previous 6 months*<br>• Severe respiratory disease with arterial oxygen saturation of less than 90% at rest<br>• Severe ischemic heart disease (exercise tolerance less than 100 yards or life expectancy <4 years) or myocardial infarction in the previous 3 months<br>• Severe inflammatory bowel disease requiring at least one hospital admission of 5 days in the last year or bowels open >6 times/day<br>* Patients answering yes to this criterion were eligible for the PPI-only (non-aspirin) randomization.	Exact definitions of what constituted 'severe' or 'frequent' for these medical conditions were added to the protocol from Protocol V10·0 dated 30 Jul 2010	Medical conditions that would make endoscopy or completing the trial difficult, including:<br><br>• Frequent transient ischemic attacks (3 or more) or severe cerebral vascular accident in the previous 6 months*<br>• Severe respiratory disease with arterial oxygen saturation of less than 90% at rest<br>• Severe ischemic heart disease (exercise tolerance less than 100 yards or life expectancy <4 years) or myocardial infarction in the previous 3 months<br>• Severe inflammatory bowel disease requiring at least one hospital admission of 5 days in the last year or bowels open >6 times/day<br>* Patients answering yes to this criterion were eligible for the PPI-only (non-aspirin) randomization.
Continuous/frequent non-steroidal anti-inflammatory drug use or COX-2 inhibitors (more than 60 days/year in total)	Unchanged	Continuous/frequent non-steroidal anti-inflammatory drug use or COX-2 inhibitors (more than 60 days/year in total)

Patients with absolute contraindications to PPIs, aspirin or their excipients i.e. allergies, ulcers, renal impairment or use of oral anticoagulants.	Unchanged	Patients with absolute contraindications to PPIs, aspirin or their excipients i.e. allergies, ulcers, renal impairment or use of oral anticoagulants.
	Added Protocol V5·0 dated 27 Jul 2005: Pregnant or lactating women	Pregnant or lactating women
	Added Protocol V6·0 dated 20 Jan 2006: Previous aspirin users will be entered providing they agree to stop aspirin use if not randomized to it	Previous aspirin users will be entered providing they agree to stop aspirin use if not randomized to it
	Added Protocol V9·0 dated 25 Sep 2007: Patients not wishing to stop aspirin or who have an absolute contraindication to it can be randomized to low/high PPI and will be analyzed for that comparison only	Patients not wishing to stop aspirin or who have an absolute contraindication to it can be randomized to low/high PPI and will be analyzed for that comparison only

Supplementary table 2: Reasons for non-enrolment in AspECT of patients meeting inclusion criteria

**Exclusion Reason**	**Number of Patients**
ALCOHOLIC	9
DECEASED	18
MISUNDERSTANDING OF TRIAL	6
NO RESPONSE	132
NO SURVEILLANCE	28
NOT MOBILE	6
ON HOLIDAY	1
ON TRIAL	22
OPTED FOR SURGERY	13
PRISONER	1
QUOTA REACHED	3
RELOCATING	26
SELF DISCHARGED	1
UNABLE TO COMPLETE FOLLOW UP	17
UNABLE TO GIVE CONSENT	8
UNSPECIFIED INELIGIBILITY	114
UNWILLING TO ADOPT TRIAL TREATMENT	13
OTHER	81
**TOTAL**	**499**

Supplementary Table 3: Baseline characteristics by treatment comparison for variables only asked of patients recruited in the first 2 years of recruitment

Variable at baseline	Low PPI N=1247	High PPI N=1244	No Aspirin N=1120	Aspirin N=1116
**BMI (kg/m^2)**	N=1254		N=1039	
median(IQR)	27 (25 , 30)	27 (25 , 30)	27 (25 , 30)	27 (25 , 30)
**Duration of Barrett's pre randomisation (years)**	N=2373		N=2123	
Median (IQR)	2·5 (0·4 , 5·7)	2·4 (0·4 , 6·1)	2·5 (0·4 , 5·9)	2·3 (0·4 , 5·8)
**Alcohol use**	N=1033		N=1032	
None	131 (25%)	125 (24%)	131 (25%)	125 (24%)
Some	385 (75%)	392 (76%)	386 (75%)	390 (76%)
(For some group, median (IQR), units per week)	10 (4 , 20)	10 (4 , 20)	10 (5 , 20)	10 (4 , 20)
**Smoker**	N=1031		N=1031	
Never	223 (43%)	223 (43%)	223 (43%)	222 (43%)
Ex	209 (41%)	201 (39%)	202 (39%)	208 (41%)
Current	84 (16%)	91 (18%)	94 (18%)	81 (16%)
**Myocardial infarction**	N=1393		N=1143	
Yes	13 (2%)	13 (2 %)	1 (0·2%)	1 (0·2%)
No	688 (98%)	679 (98%)	573 (99·8%)	568 (99·8%)
**Angina**	N=1394		N=1143	
Yes	24 (3%)	26 (4%)	3 (0·5%)	6 (1%)
No	677 (97%)	667 (96%)	572 (99·5%)	562 (99%)
**Coronary Intervention**	N=1394		N=1143	
Yes	13 (2%)	12 (2%)	0	2 (0·4%)
No	688 (98%)	681 (98%)	575 (100%)	566 (99·6%)
**Stenosis**	N=1393		N=1141	
Yes	2 (0·3%)	5 (0·7%)	0	1 (0·2%)
No	700 (99·7%)	686 (99·3%)	575 (100%)	565 (99·8%)
**Cardiac catheterisation**	N=1392		N=1140	
Yes	13 (2%)	15 (2%)	2 (0·4%)	2 (0·4%)
No	688 (98%)	676 (98%)	572 (99·6%)	564 (99·6)
**Cerebrovascular**	N=1392		N=1140	
Yes	2 (0·3%)	8 (1%)	1 (0·2%)	3 (0·5%)
No	699 (99·7%)	683 (99%)	573 (99·8%)	564 (99·5%)
**TIA**	N=1390		N=1139	
Yes	2 (0·3%)	5 (0·7%)	0	2 (0·4%)
No	696 (99·7%)	687 (99·3%)	572 (100%)	565 (99·6%)
**Peripheral Vascular Disease**	N=1378		N=1131	
Yes	6 (1%)	9 (1%)	3 (0·5%)	5 (1%)
No	686 (99%)	677 (99%)	565 (99·5%)	558 (99%)
**Diabetes**	N=1032		N=1031	
Yes	18 (3%)	13 (3%)	13 (3%)	18 (4%)
No	499 (97%)	502 (97%)	503 (97%)	497 (96%)
**Hypertension**	N=1032		N=1031	
Yes	116 (23%)	129 (25%)	122 (24%)	123 (24%)
No	399 (77%)	288 (75%)	393 (76%)	393 (76%)
**Hyperlipidaemia**	N=1034		N=1033	
Yes	47 (9%)	43 (8%)	46 (9%)	44 (9%)
No	287 (56%)	262 (51%)	275 (53%)	273 (53%)
Unknown	182 (35%)	213 (41%)	198 (38%)	197 (38%)

Supplementary Figure 1: Participant compliance with (a) PPI and (b) aspirin medication, by treatment group

Figure 1(a): PPI compliance

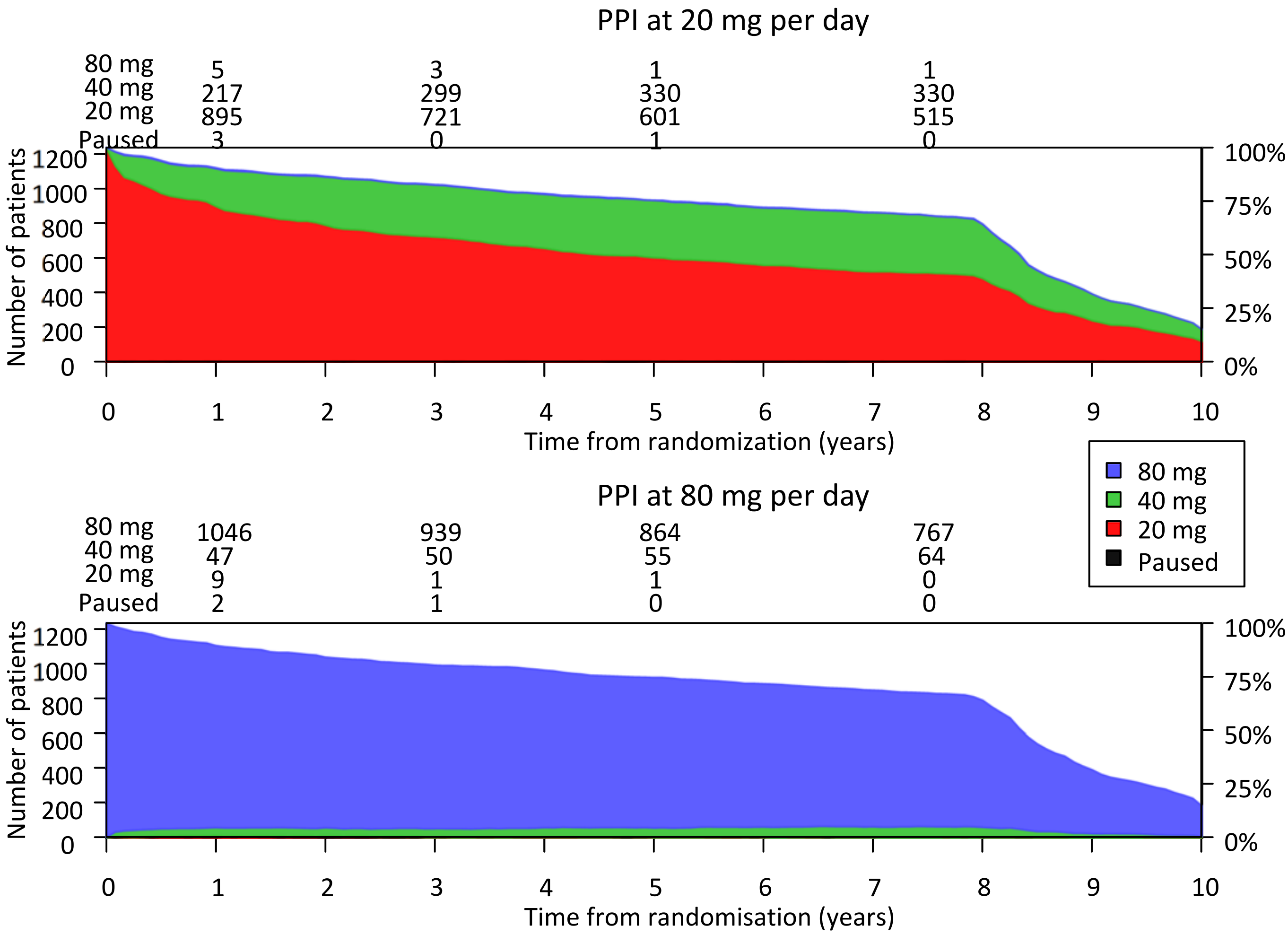

Figure 1(b): Aspirin compliance

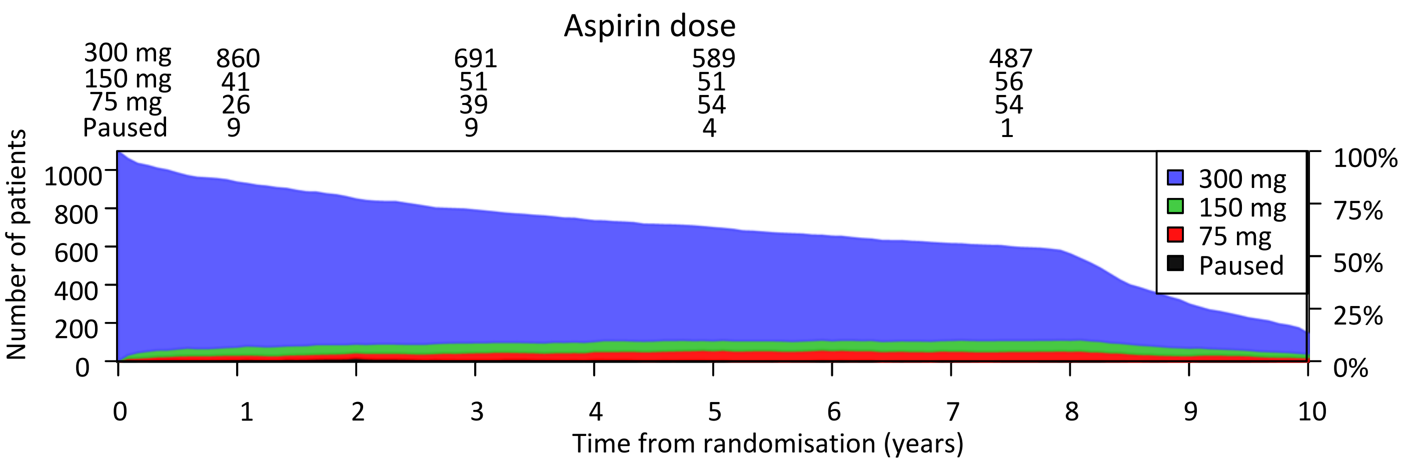

PPI and aspirin dose changes shown above were pre-specified in the protocol and permitted.

Supplementary table 4: Details of primary outcome breakdown by treatment arm

		PPI			
		High-dose		Low-dose	
Aspirin	Yes	52 / 572 = 0·091		75 / 566 = 0·133	
		All-cause mortality	25 (48%)	All-cause mortality	37 (50%)
		Oesophageal adenocarcinoma	12 (23%)	Oesophageal adenocarcinoma	19 (25%)
		High-grade dysphasia	15 (29%)	High-grade dysphasia	19 (25%)
	No	87 / 698 = 0·125		99 / 699 = 0·142	
		All-cause mortality	43 (49%)	All-cause mortality	50 (51%)
		Oesophageal adenocarcinoma	19 (22%)	Oesophageal adenocarcinoma	11 (11%)
		High-grade dysphasia	25 (29%)	High-grade dysphasia	38 (38%)

Supplementary Table 5: Primary and secondary analyses repeated using a Cox proportional hazards model

	High PPI vs low PPI				Aspirin vs no aspirin			
	Number of patients	Hazard ratio	95% CI	P value	Number of patients	Hazard ratio	95% CI	P value
**Primary aim**								
All-cause mortality or esophageal adenocarcinoma or high-grade dysplasia	2535	0·79	0·63-0·99	0·0379	2280	0·8*	0·64-1·02	0·07
**Secondary aim**								
All-cause mortality	2,535	0·74	0·55-0·99	0·0431	2,280	0·80	0·59-1·09	0·16
Esophageal adenocarcinoma	2,535	0·97	0·63-1·50	0·89	2,280	1·00	0·62-1·58	0·97
High-grade dysplasia**	2,535	0·63	0·42-0·96	0·0329	2,280	0·62	0·40-0·95	0·0283
Cause-specific mortality**	2,535	0·65	0·27-1·57	0·34	Too few events			
Males only, composite endpoint	2,022	0·79	0·62-1·01	0·06	1,796	0·79	0·61-1·02	0·08
Females only, composite endpoint	513	0·77	0·43-1·37	0·37	484	0·90	0·50-1·60	0·71

* A patient taking aspirin is estimated to be 0·8 times more likely to have an event than a patient not taking aspirin

** Competing risks Cox modelling was used for high-grade dysplasia and cause-specific mortality as the competing risk of death might have a large effect

Supplementary Table 6: Serious adverse events and serious adverse reactions by Common Terminology Criteria for Adverse Events grade

	CTCAE Grade					
**All serious adverse events by system**	**1**	**2**	**3**	**4**	**5**	**Total**
**Blood and lymphatic system disorders**	5	3	5	2	0	15
**Cardiac disorders**	9	36	77	24	12	158
**Ear and labyrinth disorders**	1	1	3	0	0	5
**Endocrine disorders**	0	0	2	0	0	2
**Eye disorders**	0	3	4	0	0	7
**Gastrointestinal disorders**	24	69	50	6	2	151
**General disorders and administration site conditions**	11	15	18	0	0	44
**Hepatobiliary disorders**	2	15	19	2	5	43
**Immune system disorders**	1	1	2	1	0	5
**Infections and infestations**	7	57	109	9	5	187
**Injury, poisoning and procedural complications**	7	23	41	4	6	81
**Investigations**	1	0	0	3	0	4
**Metabolism and nutrition disorders**	3	6	5	4	0	18
**Musculoskeletal and connective tissue disorders**	3	12	10	1	0	26
**Neoplasms benign, malignant and unspecified (including cysts and polyps)**	3	16	41	22	45	127
**Nervous system disorders**	16	41	37	13	7	114
**Psychiatric disorders**	1	3	6	4	2	16
**Renal and urinary disorders**	5	14	16	1	0	36
**Respiratory, thoracic and mediastinal disorders**	5	18	12	3	0	38
**Skin and subcutaneous tissue disorders**	0	0	1	0	0	1
**Vascular disorders**	1	5	19	6	4	35
**Total**	105	338	477	105	88	1113*
***Nineteen serious adverse events are missing a CTCAE grade.**						
**Serious adverse reactions**						
**Related to aspirin**	9	19	12	2	1	43*
**Related to esomeprazole**	2	4	10	2	1	19
**Related to both aspirin and esomeprazole**	0	2	0	0	0	2
**Total**	11	25	22	4	2	64*
***One serious adverse reaction is missing a CTCAE grade**						

CTCAE: Common Terminology Criteria for Adverse Events.

Supplementary Table 7: Total SAEs by treatment arm

	**Treatment Arm**				
**SAE System / Category**	**Low PPI**	**High PPI**	**Low PPI Asp**	**High PPI Asp**	**Total**
**Blood and lymphatic system disorders**	2	5	9	1	17
**Cardiac disorders**	37	50	38	35	160
**Ear and labyrinth disorders**	0	1	1	3	5
**Endocrine disorders**	1	0	0	1	2
**Eye disorders**	0	2	1	4	7
**Gastrointestinal disorders**	39	34	41	38	152
**General disorders and administration site conditions**	8	16	8	15	47
**Hepatobiliary disorders**	10	8	14	11	43
**Immune system disorders**	1	3	0	1	5
**Infections and infestations**	49	46	35	58	188
**Injury, poisoning and procedural complications**	18	26	24	14	82
**Investigations**	1	1	1	1	4
**Metabolism and nutrition disorders**	5	8	1	4	18
**Musculoskeletal and connective tissue disorders**	9	2	6	9	26
**Neoplasms benign, malignant and unspecified (incl cysts and polyps)**	38	41	28	27	134
**Nervous system disorders**	30	27	31	26	114
**Pregnancy, puerperium and perinatal conditions**	1	0	0	0	1
**Psychiatric disorders**	4	6	1	5	16
**Renal and urinary disorders**	14	9	5	8	36
**Respiratory, thoracic and mediastinal disorders**	9	6	18	6	39
**Skin and subcutaneous tissue disorders**	0	1	0	0	1
**Vascular disorders**	7	11	10	7	35
**OVERALL TOTAL**	**283**	**303**	**272**	**274**	**1132**

Supplementary Table 8: Gastrointestinal and non-gastrointestinal bleeds classified as serious adverse events in each treatment group.

	**Low or high PPI**		**Aspirin or no aspirin**	
**Serious adverse events by system / category**	Low PPI	High PPI	Aspirin	No aspirin
**Gastrointestinal bleeds (CTCAE grade 3–5 bleeds)**				
**Colonic hemorrhage**	1 (0)	0 (0)	0 (0)	1 (0)
**Duodenal hemorrhage**	0 (0)	2 (1)	2 (1)	0 (0)
**Esophageal hemorrhage**	3 (1)	1 (0)	3 (1)	1 (0)
**Esophageal varices hemorrhage**	0 (0)	1 (1)	0 (0)	1 (1)
**Gastric hemorrhage**	3 (1)	3 (1)	4 (1)	1 (1)
**Hemorrhoidal hemorrhage**	2 (1)	1 (0)	3 (1)	0 (0)
**Rectal hemorrhage**	1 (0)	2 (0)	2 (0)	1 (0)
**Upper gastrointestinal hemorrhage**	3 (0)	5 (1)	4 (1)	1 (0)
**Total**	13 (3)	15 (4)	18 (5)	6 (2)
**Non-gastrointestinal bleeds (CTCAE grade 3–5)**				
**Postoperative hemorrhage**	3 (3)	2 (0)	3 (3)	2 (0)
**Intracranial hemorrhage**	5 (4)	5 (2)	5 (4)	5 (4)
**Hematuria**	3 (0)	3 (2)	2 (2)	1 (0)
**Epistaxis**	11 (1)	4 (0)	13 (2)	2 (0)
**Total**	22 (8)	14 (4)	23 (11)	10 (4)
**Overall total**	35 (11)	29 (9)	41 (14)	16 (6)

Presented as total bleeds in each category (grade 3–5 bleeds in that category).

CTCAE: Common Terminology Criteria for Adverse Events

Supplementary Table 9: Details of Gastrointestinal and non-gastrointestinal bleeds by treatment arm

	Treatment Arm				
**SAE System / Category**	**Low PPI**	**High PPI**	**Low PPI Asp**	**High PPI Asp**	**Total**
**Gastrointestinal disorders**					
**Colonic hemorrhage**	1	0	0	0	1
**Duodenal hemorrhage**	0	0	0	2	2
**Esophageal hemorrhage**	1	0	2	1	4
**Esophageal varices hemorrhage**	0	1	0	0	1
**Gastric hemorrhage**	1	1	2	2	6
**Hemorrhoidal hemorrhage**	0	0	2	1	3
**Rectal hemorrhage**	1	0	0	2	3
**Upper gastrointestinal hemorrhage**	1	3	2	2	8
**Total**	**5**	**5**	**8**	**10**	**28**
**Injury, poisoning and procedural complications**					
**Postoperative hemorrhage**	0	2	3	0	5
**Total**	**0**	**2**	**3**	**0**	**5**
**Nervous system disorders**					
**Intracranial hemorrhage**	3	2	2	3	10
**Total**	**3**	**2**	**2**	**3**	**10**
**Renal and urinary disorders**					
**Hematuria**	3	1	0	2	6
**Total**	**3**	**1**	**0**	**2**	**6**
**Respiratory, thoracic and mediastinal disorders**					
**Epistaxis**	2	0	9	4	15
**Total**	**2**	**0**	**9**	**4**	**15**
**OVERALL TOTAL**	**13**	**10**	**22**	**19**	**64**

Supplementary Table 10: Primary analyses by age group

	Number of patients	Time ratio (TR)	95% CI	P value
**<60**				
Aspirin vs no aspirin	1264	1.22	0.82 , 1.81	0.326
High PPI vs low PPI	1341	1.22	0.84 , 1.79	0.296
**60+**				
Aspirin vs no aspirin	1016	1.26	0.94 , 1.69	0.118
High PPI vs low PPI	1194	1.30	0.98 , 1.71	0.064

Supplementary Table 11: Details of numbers with LGD at baseline and newly diagnosed at follow up

	Arm A	Arm B	Arm C	Arm D	Total
LGD at baseline	31	15	11	14	71
LGD diagnosed at follow up	72	60	61	56	249

Supplementary table 12: Primary analyses by treatment withdrawal or completion

	Number of patients	Time ratio (TR)	95% CI	P value
**Withdrawn Treatment Early**				
Aspirin vs no aspirin	866	1.22	0.95 , 1.56	0.114
High PPI vs low PPI	975	1.20	0.95 , 1.52	0.125
**Completed Treatment**				
Aspirin vs no aspirin	1414	1.73	0.76 , 3.96	0.192
High PPI vs low PPI	1560	1.11	0.51 , 2.44	0.787

Supplementary table 13: Cardiac Disorder Details

Details of cardiac disorders by aspirin allocation

Cardiac Disorder	Aspirin	No Aspirin
**Acute coronary syndrome**	**5**	**4**
**Aortic stenosis**	**1**	
**Aortic valve disease**	**1**	**1**
**Atrial fibrillation**	**2**	**4**
**Atrioventricular block complete**	**2**	
**Cardiac arrest**	**1**	**2**
**Cardiomyopathy**	**2**	**3**
**Chest pain - cardiac**	**5**	**4**
**Heart failure**	**5**	**2**
**Myocardial infarction**	**21**	**21**
**Pericardial effusion**	**1**	**1**
**Sinus bradycardia**	**6**	
**Ventricular tachycardia**	**1**	
	**53**	**42**

Supplementary table 14. Primary analysis by gender

	Number of patients	Time ratio (TR)	95% CI	P value
**Men**				
Aspirin vs no aspirin	1,796	1.26	0.98 , 1.64	0.074
High PPI vs low PPI	2,022	1.26	0.99 , 1.61	0.059
**Women**				
Aspirin vs no aspirin	484	1.13	0.63 , 2.02	0.687
High PPI vs low PPI	513	1.27	0.72 , 2.27	0.411

Supplementary table 15: Baseline of AspECT trial participants, stratified by randomised group

Variable at baseline	Low PPI no aspirin N=699	High PPI no aspirin N=698	Low PPI and aspirin N=566	High PPI and aspirin N=572	TOTAL
**Maximum Length of Barrett's metaplasia at randomisation (cm)** median (IQR)	4 (3 , 6)	4 (2 , 6)	4 (3 , 6)	4 (3 , 6)	**2,413**
**Length of Barrett's (stratification group)**					**2,535**
**<2cm**	69 (10%)	69 (10%)	54 (9%)	55 (9%)	
**2-3cm**	237 (34%)	237 (34%)	197 (35%)	198 (35%)	
**3-8cm**	293 (42%)	291 (42%)	245 (43%)	248 (43%)	
**>8cm**	71 (10%)	70 (10%)	59 (10%)	59 (10%)	
**Tongues**	29 (4%)	31 (5%)	11 (2%)	12 (2%)	
**Age (years)** (median (IQR))	59 (51 , 65)	59 (51 , 66)	58 (50 , 64)	58 (50 , 65)	**2,535**
**Age (stratification grouping)**					**2,535**
**<50 years**	148 (21%)	143 (21%)	135 (24%)	137 (24%)	
**50-60 years**	210 (30%)	210 (30%)	178 (31%)	180 (31%)	
**60-70 years**	252 (36%)	252 (36%)	195 (35%)	193 (34%)	
**>70 years**	89 (13%)	93 (13%)	58 (10%)	62 (11%)	
**Sex**					**2,535**
Male	564 (81%)	562 (81%)	448 (79%)	448 (78%)	
Female	135 (19%)	136 (19%)	118 (21%)	124 (22%)	
**Intestinal metaplasia (stratification group)**					**2,535**
Yes	616 (88%)	615 (88%)	514 (91%)	521 (91%)	
No	83 (12%)	83 (12%)	52 (9%)	51 (9%)	

Supplementary table 16: Serious adverse events CTCAE grade 3-5 by treatment arm

System affected by serious adverse event	Arm A N=1265	Arm B N=1270	Arm C N=1142	Arm D N=1138
**Serious adverse events**				
Blood and lymphatic system disorders		3	4	
Cardiac disorders	29	31	28	25
Ear and labyrinth disorders		1	1	1
Endocrine disorders	1			1
Eye disorders		1	1	2
Gastrointestinal disorders	15	11	15	17
General disorders and administration site conditions	5	5	2	6
Hepatobiliary disorders	8	6	8	4
Immune system disorders	1	2		
Infections and infestations	32	27	25	39
Injury, poisoning and procedural complications	13	14	15	9
Investigations	1		1	1
Metabolism and nutrition disorders	2	5		2
Musculoskeletal and connective tissue disorders	4		3	4
Neoplasms benign, malignant and unspecified (including cysts and polyps)	33	34	23	18
Nervous system disorders	16	13	15	13
Psychiatric disorders	3	4	1	4
Renal and urinary disorders	4	5	3	5
Respiratory, thoracic and mediastinal disorders	3	4	5	3
Skin and subcutaneous tissue disorders		1		
Vascular disorders	6	9	9	5
**Total**	**176**	**176**	**159**	**159**
**Serious adverse reactions**				
Related to aspirin	0	0	6	1
Related to esomeprazole	4	4	0	2
Related to both aspirin & esomeprazole	0	0	0	0
**Total**	**4**	**4**	**6**	**3**

Supplementary table 17: Inclusion criteria for per protocol population

**Treatment**	**Therapeutic dose**
Low PPI no aspirin<br><br>High PPI no aspirin	1 year of esomeprazole at randomised dose<br>OR<br>event before 1 year and esomeprazole at randomised dose until the event
Low PPI + aspirin<br><br>High PPI + aspirin	1 year of esomeprazole at randomised dose and at least 6 months of aspirin at randomised dose<br>OR<br>event before 6 months and esomeprazole and aspirin at randomised dose until the event<br>OR<br>event between 6 and 12 months and esomeprazole at randomised dose until the event and aspirin at randomised dose for at least 6 months

Supplementary table 18: Accelerated failure time per protocol analysis for both primary comparisons

	**Number of patients**	**Time ratio (TR)**	**95% CI**	**P value**
Aspirin vs no aspirin	1,812	1.25	0.96 , 1.63	0.101
High PPI vs low PPI	2,008	1.16	0.90 , 1.48	0.252

Supplementary figure 2: Kaplan Meier curves for comparison of Aspirin vs no Aspirin and high dose PPI vs low dose PPI

2 (a). Aspirin and HGD/Adenocarcinoma:

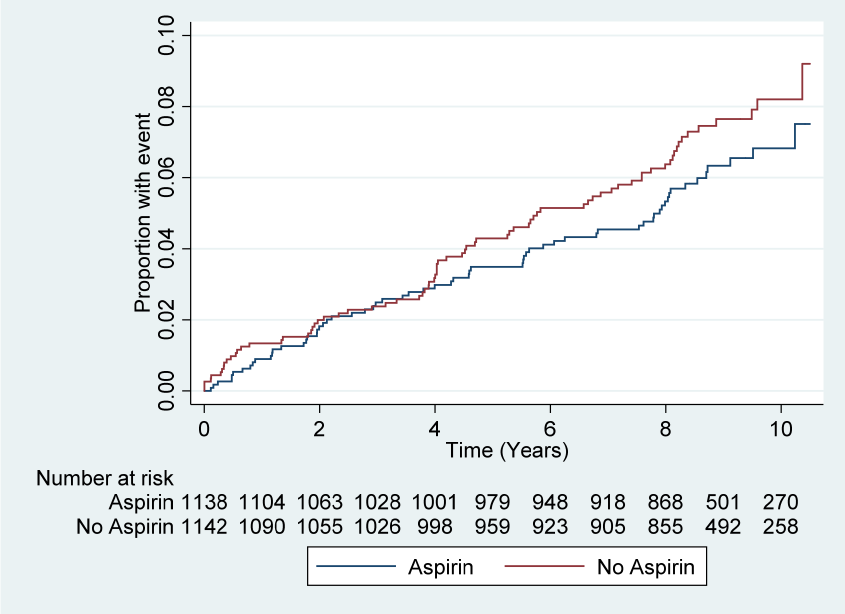

2(b): Aspirin and all-cause mortality:

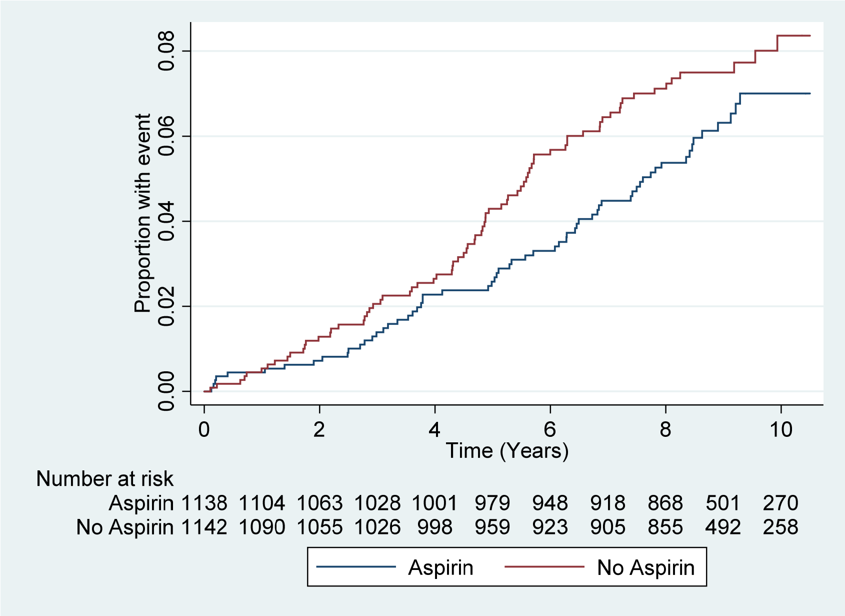

2(c) PPI and HGD/Adenocarcinoma:

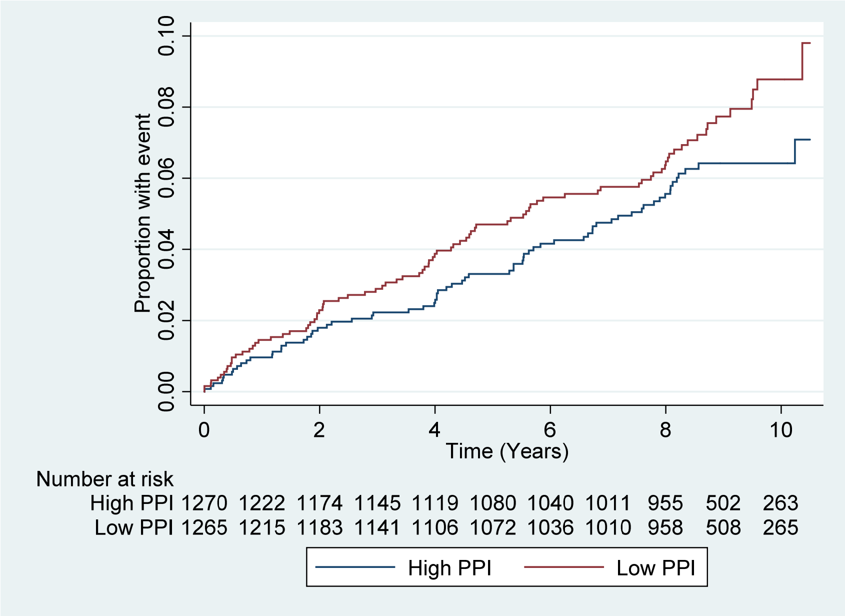

2 (d) PPI and all-cause mortality:

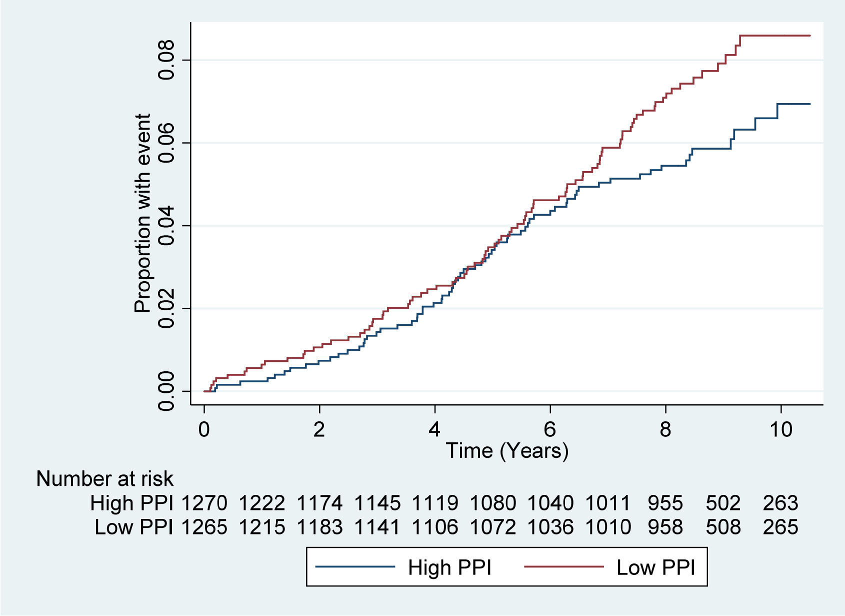

Supplement: Supplementary appendix [file mmc1.pdf]
